# Bending analysis of sandwich panel composite with a re-entrant lattice core using zig-zag theory

**DOI:** 10.1038/s41598-022-19930-x

**Published:** 2022-09-22

**Authors:** M. J. Khoshgoftar, A. Barkhordari, M. Limuti, F. Buccino, L. Vergani, M. J. Mirzaali

**Affiliations:** 1 Department of Mechanical Engineering, Faculty of Engineering, Arak University, Arak, 3815688349 Iran; 2 Department of Mechanical Engineering, Politecnico Di Milano, Via La Masa, 1, 20156 Milan, Italy; 3 Department of Biomechanical Engineering, Faculty of Mechanical, Maritime, and Materials Engineering, Delft University of Technology (TU Delft), Mekelweg 2, 2628 CD Delft, The Netherlands

**Keywords:** Aerospace engineering, Biomedical engineering, Civil engineering, Mechanical engineering

## Abstract

The sandwich panel structures have been widely used in many industrial applications because of their high mechanical properties. The middle layer of these structures is very important factor in controlling and enhancing their mechanical performance under various loading scenarios. The re-entrant lattice configurations, are prominent candidates that can be used as the middle layer in such sandwich structures because of several reasons namely the simplicity in tuning their elastic (*e.g.*, values of Poisson’s ratio and elastic stiffness) and plastic (*e.g.*, high strength-to-weight ratio) properties by only adjusting the geometrical features of the constituting unit cells. Here, we investigated the response of a three-layered sandwich plate with a re-entrant core lattice under flexural bending using analytical (*i.e.*, zig-zag theory), computational (*i.e.*, finite element) and experimental tests. We also analyzed the effects of different geometrical parameters (*e.g.*, angle, thicknesses, and length to the height ratio of unit cells) of re-entrant lattice structures on the overall mechanical behavior of sandwich structures. We found that the core structures with auxetic behavior (*i.e.*, negative Poisson’s ratio) resulted in a higher bending strength and a minimum out-of-plane shear stress as compared to those with conventional lattices. Our results can pave way in designing advanced engineered sandwich structures with architected core lattices for aerospace and biomedical applications.

## Introduction

The sandwich structures have been widely used in many industries such as machine and sport equipment designs, marine, aerospace, and biomedical engineering due to their high strength and low weight properties. The re-entrant lattice structures are among the potential candidates to be considered as a core layer in such composite structures because of their excellent energy absorption capacity and high strength to weight properties^1–3^. Significant efforts have been made in the past to design lightweight sandwich structures with re-entrant lattices to obtain even more improved mechanical properties. Examples of those structures are high-pressure loading in ship hulls, and shock absorbers in automobiles^4,5^. What makes the re-entrant lattice structures extremely popular, unique and suitable for the sandwich panel designs is the ability to tune their elastic mechanical properties (*i.e.*, elastic stiffness and Poisson’s ratio) independently by simply adjusting their microstructural geometries at the smaller scale. Among these interesting properties is the auxetic behavior (or negative Poisson’s ratio) which refers to a transverse expansion of the lattice structures, when they are longitudinally stretched^6^. This unusual behavior originates from the microstructural design of their constituting unit cells^7–9^.

After initial studies by Lakes about the production of auxetic foams, significant efforts have been made to design porous structures with negative values of Poisson’s ratio^10,11^. Towards this aim, several geometrical designs have been proposed such as chiral, semi-rigid and rigid rotating unit cells^12^ all of which exhibiting auxetic behavior. The advent of additive manufacturing (AM, also known as 3D printing) techniques has also helped the realization of these 2D or 3D auxetic structures^13^.

The auxetic behavior offers unique mechanical properties. For example, Lakes and Elms^14^ showed that the auxetic foams have higher yield strengths, higher energy absorption capacity against the impact loading, and lower stiffness properties as compared to the conventional foams. As far as the dynamic mechanical properties of the auxetic foams are concerned, they showed higher resilience under dynamic crushing loads and higher elongation capacity under the pure stretching^15^. In addition, the use of auxetic fibers as reinforcement in composites would result in enhancing their mechanical properties^16^ and their resistance to the damage originated from the fiber elongations^17^.

It has also been shown that the use of re-entrant auxetic structure as a core in curved composite structures could increase their out-of-plane properties, including flexural stiffness and strength^18^. Using a delamination model, it has also been observed that the auxetic core could increase the fracture strength of the composite plates^19^. The composites with auxetic fibers could also prevent the propagation of cracks in comparison to those with conventional fibers^20^.

Zhang et al.^21^ simulated the dynamic crashing behaviors of re-entrant cellular structures. They found that the stress and energy absorption could be improved by increasing the angle of auxetic unit cells resulting in a lattice with a more negative values of Poisson’s ratio. They also suggested that such auxetic sandwich panels could be used as a protective structure against the high-strain rate impact loads. Imbalzano et al.^22^ also reported that auxetic composite panels could dissipate more energy (*i.e.*, two times higher) via a plastic deformation and could reduce up to 70% of the back facet's maximum velocity compared to a single-layer panel.

Recently, the numerical and experimental investigations of the auxetic core sandwich structures have gained a lot of attentions. These studies have highlighted the ways on how to improve the mechanical properties of those sandwich structures. As an example, considering sufficiently thick auxetic layer as a core in a sandwich panel, could lead to a higher effective Young's modulus than the Young's modulus of its constituting stiffest layer^23^. Also, using optimization algorithms, the bending performance of sandwich beams^24^ or tubular lattices^25^ with auxetic cores could be improved. There are also other studies concerning mechanical testing of auxetic core sandwich structures under more complex loading scenarios. Examples are compressive testing of concrete composites with auxetic cores^26^, sandwich panels in a blast loading^27^, flexural bending^28^, and low-velocity impact resistance tests^29^, and nonlinear bending analysis of sandwich plates with functionally graded auxetic cores^30^.

Since the computational simulations and experimental evaluations of such structures are often very time-consuming and expensive, there is a need for the development of theoretical approaches that can effectively and accurately provide the required information for designing a core auxetic sandwich structure under arbitrary loading conditions in a reasonable time. The current analytical approaches, however, suffer from many limitations. These theories in particular, are not accurate enough for predicting the behavior of relatively thick composites and for analyzing composites that are composed of several materials with highly dissimilar elastic properties.

Since these analytical models depend on the applied loads and boundary conditions, here, we focus on the bending properties of sandwich panels with an auxetic core. The equivalent single-layer theories for such analyses fall short of correctly predicting the shear and axial stresses in highly heterogeneous lay-ups in moderately thick sandwich composites. Moreover, the number of kinematic variables (*e.g.*, displacement, velocity etc*.*) in some theories such as layer-wise theories, highly depends on the number of layers. This means that the kinematic fields of each layer can independently be described while satisfying certain physical continuity constraints. Therefore, this will end up in considering a great number of variables in the model, making such approaches computationally very expensive. To overcome these limitations, we propose a method based on the zig-zag theory that is a particular sub-class of layer-wise theory. This theory enforces the continuity of the shear stresses across the entire laminate thickness by assuming a zig-zag pattern for the in-plane displacements. The zig-zag theory, therefore, gives the same number of kinematic variables regardless of the number of layers in a laminate.

In order to show the capacity of our approach in predicting the behavior of sandwich panels with re-entrant cores under bending loading, we compared our results with the classical theories (*i.e.*, 3D elasticity (Pagano), first-order shear deformation theory (FSDT)) of plates and validated our approach by computational models (*i.e.*, finite element) and experimental data (*i.e.*, three-point bending of the 3D printed sandwich panels). For this purpose, we, first, derived the displacement relations based on the zig-zag theory, and then, obtained the governing equations using Hamilton's principle and solved them by the Galerkin method. Our results suggested a powerful tool for designing the corresponding geometrical parameters of a sandwich panel with an auxetic core helping in finding structures with improved mechanical properties.

## Zig-zag formulation

**Figure 1:**
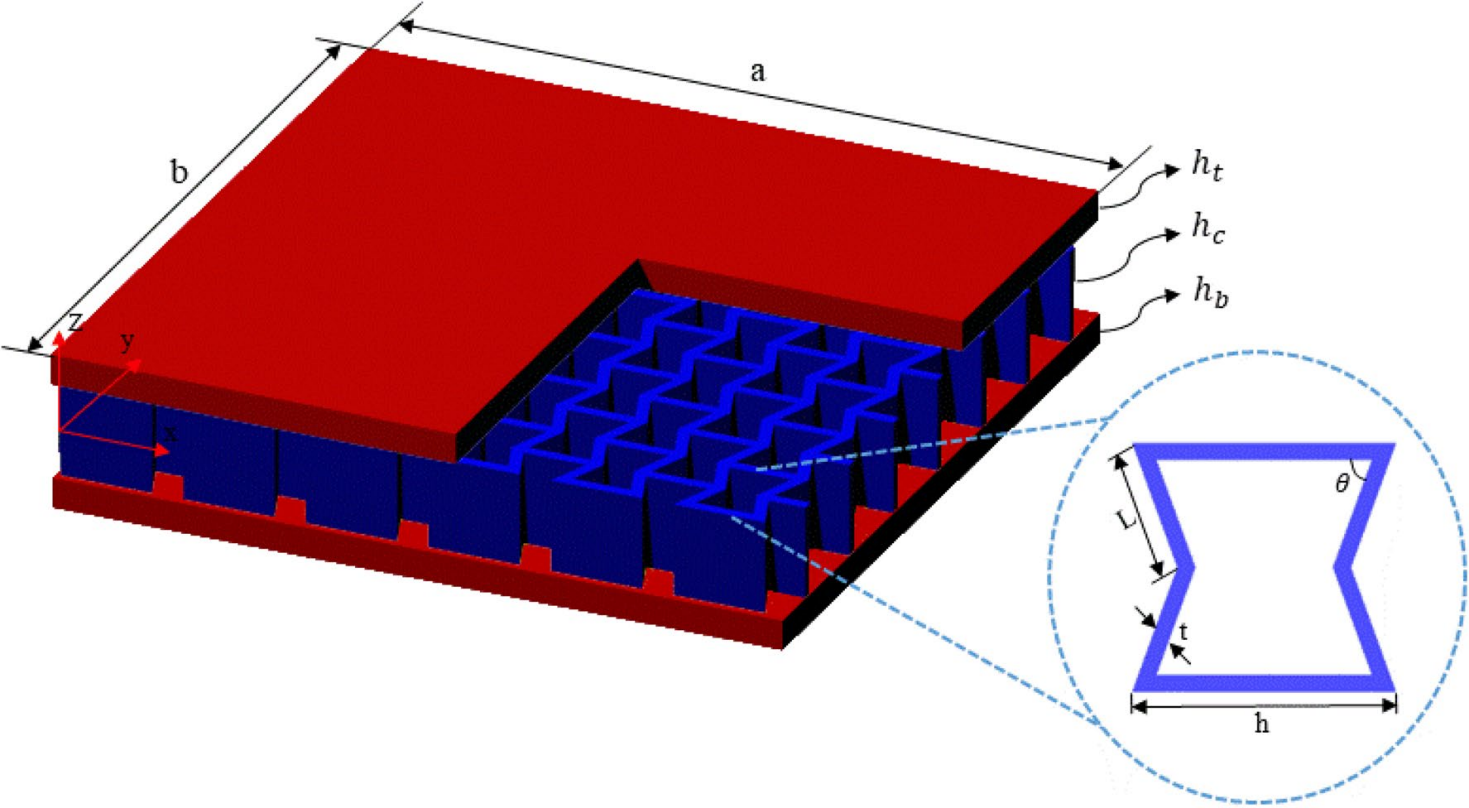
Three-layered sandwich plate with a re-entrant lattice structure as a core.

Let us consider a three-layer sandwich plate (Fig. 1). The geometric parameters of this structure are: top layer, ${h}_{t}$, middle layer, ${h}_{c}$, and, bottom layer, ${h}_{b}$ thicknesses. We assume that the structural core is composed of a re-entrant lattice structure. This structure is composed of unit cells that are arranged in an ordered manner next to each other. By changing the geometrical parameters of the re-entrant structure their mechanical properties (*i.e.*, values of Poisson’s ratio and elastic stiffness) can be changed. The geometrical parameters of a unit cell, as shown in Fig. 1, are angle (θ), length (h), height (L), and strut thickness (t).

The zig-zag theory provides a very accurate prediction of the stress and strain behaviors of moderately thick layered composite structures. The structure displacement in a zig-zag theory consists of two parts. The first part shows the behavior of the whole sandwich plate, and the second part considers the behavior between the layers so that to satisfy the shear stress continuity (or so-called zig-zag functions). Also, the zig-zag function vanishes on the outer surfaces of the laminated plate instead of within a given layer. As a result, the zig-zag function provides a contribution from each layer to the overall deformation of the cross-section. This important difference ensures a more physically realistic distribution for the zig-zag function instead of other zig-zag functions. The present refined zig-zag model does not enforce the continuity of the transverse shear stresses along the intermediate layers. The displacement field based on the zig-zag theory can, therefore, be written as follows^31^.

1 $$\begin{aligned} U^{k} \left( {x,y,z,t} \right) = & u\left( {x,y,t} \right) + {\rm{z}}{\uptheta}_{x} \left( {x,y,t} \right) + \phi _{x}^{k} \left( z \right)\psi _{x} \left( {x,y,t} \right) \\ V^{k} \left( {x,y,z,t} \right) = & v\left( {x,y,t} \right) + {\rm{z}}{\uptheta}_{y} \left( {x,y,t} \right) + \phi _{y}^{k} \left( z \right)\psi _{y} \left( {x,y,t} \right) \\ W^{k} \left( {x,y,z,t} \right) = & w\left( {x,y,t} \right) \\ \end{aligned}$$

In Eq. (1), *k* = *b, c, t* represents the bottom, middle, and top layer, respectively. The displacement field of the midplane along the Cartesian axis (x,y,z) is (u,v,w), and in-plane bending rotation about the (x,y) axis is ${\uptheta }_{x}$ and ${\uptheta }_{y}$. ${\psi }_{x}$ and ${\psi }_{y}$ are the spatial amplitudes of the zig-zag rotation, while ${\phi }_{x}^{k}\left(z\right)$ and ${\phi }_{y}^{k}\left(z\right)$, represent zig-zag functions.

The zig-zag amplitudes are vector functions of the actual response of the plate under the applied loading. They provide the proper scaling of the zig-zag functions, thus controlling the total zig-zag contribution to the in-plane displacements. The shear strain along the thickness of the plate consists of two parts. The first part is the uniform shear angle through the total laminate thickness, and the second part is the piecewise constant functions that are uniform through the thickness of each individual layer. According to these piecewise constant functions, the zig-zag function for each layer can be written as:

2 $$\begin{gathered} \phi_{x}^{b} = \left( {z + h} \right)\left( {\frac{{G_{x} }}{{c_{44}^{b} }} - 1} \right) \hfill \\ \phi_{y}^{b} = \left( {z + h} \right)\left( {\frac{{G_{y} }}{{c_{55}^{b} }} - 1} \right) \hfill \\ \phi_{x}^{c} = \left( {z + h} \right)\left( {\frac{{G_{x} }}{{c_{44}^{c} }} - 1} \right) + 2h_{b} \left( {\frac{{G_{y} }}{{c_{44}^{b} }} - \frac{{G_{y} }}{{c_{44}^{c} }}} \right) \hfill \\ \phi_{y}^{c} = \left( {z + h} \right)\left( {\frac{{G_{y} }}{{c_{55}^{c} }} - 1} \right) + 2h_{b} \left( {\frac{{G_{y} }}{{c_{55}^{b} }} - \frac{{G_{y} }}{{c_{55}^{c} }}} \right) \hfill \\ \phi_{x}^{t} = \left( {z + h} \right)\left( {\frac{{G_{x} }}{{c_{44}^{t} }} - 1} \right) + 2h_{b} \left( {\frac{{G_{x} }}{{c_{44}^{b} }} - \frac{{G_{x} }}{{c_{44}^{t} }}} \right) + 2h_{c} \left( {\frac{{G_{x} }}{{c_{44}^{c} }} - \frac{{G_{x} }}{{c_{44}^{t} }}} \right) \hfill \\ \hfill \\ \phi_{y}^{t} = \left( {z + h} \right)\left( {\frac{{G_{y} }}{{c_{55}^{t} }} - 1} \right) + 2h_{b} \left( {\frac{{G_{y} }}{{c_{55}^{b} }} - \frac{{G_{y} }}{{c_{55}^{t} }}} \right) + 2h_{c} \left( {\frac{{G_{y} }}{{c_{55}^{c} }} - \frac{{G_{y} }}{{c_{55}^{t} }}} \right) \hfill \\ \end{gathered}$$

In Eq. (2),${c}_{11}^{k}$ and ${c}_{22}^{k}$ are the elastic constants of each layer, and h is the total thickness of the plate. Also, ${G}_{x}$ and ${G}_{y}$ are weighted-average transverse-shear stiffness coefficients, which are represented as^31^:

3 $$\begin{gathered} G_{x} = \left( {\frac{1}{2h}\mathop \smallint \limits_{ - h}^{h} \frac{dz}{{c_{11}^{k} }}} \right)^{ - 1} \hfill \\ G_{y} = \left( {\frac{1}{2h}\mathop \smallint \limits_{ - h}^{h} \frac{dz}{{c_{22}^{k} }}} \right)^{ - 1} \hfill \\ \end{gathered}$$

The two zig-zag amplitude functions (Eq. (3)) and the remaining five kinematic variables (Eq. (2)) of first-order shear deformation theory constitute a set of seven kinematic variables associated with this refined zig-zag plate theory. By assuming linear strain relations, the strain field in the Cartesian coordinate system with considering the zig-zag theory can be obtained as:

4 $$\begin{gathered} \varepsilon_{{{\text{xx}}}}^{k} = \frac{\partial u}{{\partial x}} + z\left( {\frac{{\partial \theta_{x} }}{\partial x}} \right) + \phi_{x}^{k} \frac{{\partial \psi_{x} }}{\partial x} \hfill \\ \varepsilon_{{{\text{yy}}}}^{k} = \frac{\partial v}{{\partial y}} + z\left( {\frac{{\partial \theta_{y} }}{\partial y}} \right) + \phi_{y}^{k} \frac{{\partial \psi_{y} }}{\partial y} \hfill \\ \gamma_{{{\text{xy}}}}^{k} = \frac{\partial u}{{\partial y}} + \frac{\partial v}{{\partial x}} + z\left( {\frac{{\partial \theta_{x} }}{\partial y} + \frac{{\partial \theta_{x} }}{\partial x}} \right) + \phi_{x}^{k} \frac{{\partial \psi_{x} }}{\partial y} + \phi_{y}^{k} \frac{{\partial \psi_{y} }}{\partial x} \hfill \\ \gamma_{{{\text{xz}}}}^{k} = \frac{\partial w}{{\partial x}} + \theta_{x} + \frac{{\partial \phi_{x}^{k} }}{\partial z}\psi_{x} \hfill \\ \gamma_{{{\text{yz}}}}^{k} = \frac{\partial w}{{\partial y}} + \theta_{y} + \frac{{\partial \phi_{y}^{k} }}{\partial z}\psi_{y} \hfill \\ \end{gathered}$$

where ${\varepsilon }_{yy}$ and ${\varepsilon }_{xx}$ are normal strains and ${\gamma }_{yz},{ \gamma }_{xz}$ and ${\gamma }_{xy}$ are shear strains.

Using Hooke's law and considering the zig-zag theory, the stress-strain relationships for an orthotropic plate with a re-entrant lattice structure can be obtained by Eq. (5)^32^ where ${c}_{ij}$ is the elastic constants of the stress-strain matrix.

5 $$\begin{gathered} \left[ {\begin{array}{*{20}c} {\sigma_{xx}^{c} } \\ {\sigma_{{{\text{yy}}}}^{c} } \\ {\sigma_{{{\text{xy}}}}^{c} } \\ \end{array} } \right] = \left[ {\begin{array}{*{20}c} {c_{11}^{c} } & {c_{12}^{c} } & 0 \\ {c_{12}^{c} } & {c_{22}^{c} } & 0 \\ 0 & 0 & {c_{66}^{c} } \\ \end{array} } \right]\left[ {\begin{array}{*{20}c} {\varepsilon_{{{\text{xx}}}}^{c} } \\ {\varepsilon_{{{\text{yy}}}}^{c} } \\ {\gamma_{{{\text{xy}}}}^{c} } \\ \end{array} } \right] \hfill \\ \left[ {\begin{array}{*{20}c} {\sigma_{yz}^{c} } \\ {\sigma_{xz}^{c} } \\ \end{array} } \right] = \left[ {\begin{array}{*{20}c} {c_{44}^{c} } & 0 \\ 0 & {c_{55}^{c} } \\ \end{array} } \right]\left[ {\begin{array}{*{20}c} {\gamma_{{{\text{yz}}}}^{c} } \\ {\gamma_{{{\text{xz}}}}^{c} } \\ \end{array} } \right] \hfill \\ \end{gathered}$$

By considering orthotropic material models, the elastic constants can be calculated as:

6 $$\begin{gathered} c_{11}^{k} = \frac{{E_{11}^{k} }}{{1 - v_{12}^{k} v_{21}^{k} }} \hfill \\ c_{12}^{k} = \frac{{E_{22}^{k} v_{12}^{k} }}{{1 - v_{12}^{k} v_{21}^{k} }} \hfill \\ c_{22}^{k} = \frac{{E_{22}^{k} }}{{1 - v_{12}^{k} v_{21}^{k} }} \hfill \\ c_{44}^{k} = G_{23}^{k} \hfill \\ c_{55}^{k} = G_{13}^{k} \hfill \\ c_{66}^{k} = G_{12}^{k} \hfill \\ \end{gathered}$$

where ${G}_{ij}^{k}$، ${E}_{ij}^{k}$ and ${v}_{ij}^{k}$ are shear modulus, Young's modulus, and Poisson's ratios in different directions, respectively. These coefficients are equal for the isotopic layers in all directions. Also, for a re-entrant lattice core, as shown in Fig. 1, these properties can be re-written as^33^.

7 $$\begin{gathered} E_{11}^{c} = E^{c} \left( \frac{t}{l} \right)^{3} \frac{{\left( {\frac{h}{l} - \cos \theta } \right)}}{{\left( {1 + \left( {\cot^{2} \theta + \frac{h}{l}\csc^{2} \theta } \right)\left( \frac{t}{l} \right)^{2} } \right)\sin^{3} \theta }} \hfill \\ E_{22}^{c} = E^{c} \left( \frac{t}{l} \right)^{3} \frac{1}{{\sin \theta \left( {\frac{h}{l} - \cos \theta } \right)\left( {\cot^{2} \theta + \left( \frac{t}{l} \right)^{2} } \right)}} \hfill \\ v_{12}^{c} = - \frac{{\cos \theta \left( {1 - \left( \frac{t}{l} \right)^{2} } \right)\left( {\frac{h}{l} - \cos \theta } \right)}}{{\left( {\sin^{2} \theta } \right)\left( {1 + \left( {\left( {\cot^{2} \theta + \csc^{2} \theta } \right)\left( \frac{h}{l} \right)} \right)\left( \frac{t}{l} \right)^{2} } \right)}} \hfill \\ G_{12}^{c} = E^{c} \left( \frac{t}{l} \right)^{3} \frac{1}{{\left( \frac{h}{l} \right)\left( {1 + 2\frac{h}{l}} \right)\sin \theta }} \hfill \\ G_{13}^{c} = G^{c} \left( {\frac{{\left( \frac{t}{l} \right)}}{2\sin \theta }} \right)\left( {\frac{{\frac{h}{l} - \cos \theta }}{{1 + 2\frac{h}{l}}} + \frac{{\frac{h}{l} + 2\cos^{2} \theta }}{{2\left( {\frac{h}{l} - \cos \theta } \right)}}} \right) \hfill \\ G_{23}^{c} = G^{c} \left( \frac{t}{l} \right)\frac{\sin \theta }{{\left( {\frac{d}{l} - \cos \theta } \right)}} \hfill \\ \end{gathered}$$

By applying the Hamilton principle to the equation of motions of sandwich plate with a re-entrant lattice core, the governing equations of the structure can be obtained. The Hamilton principle is written as:

8 $$\mathop \smallint \limits_{0}^{T} \left( {{\delta U} - {\updelta }W} \right){\text{d}}t = 0$$

where *δ* represents the variation operator, *U* represents the strain potential energy, and *W* is the work done by the external forces. The total strain potential energy is obtained using Eq. (9), where *A* is the midplane domain.

9 $${\rm{\delta }}U = \mathop \smallint \limits_{{\rm{A}}} \mathop \smallint \limits_{{\frac{{ - h}}{2}}}^{{\frac{h}{2}}} \left( {\sigma _{{{\rm{xx}}}}^{k} {\rm{\delta }}\varepsilon _{{{\rm{xx}}}}^{k} + \sigma _{{{\rm{yy}}}}^{k} {\rm{\delta }}\varepsilon _{{{\rm{yy}}}}^{k} + \tau _{{{\rm{xy}}}}^{k} {\rm{\delta \gamma }}_{{{\rm{xy}}}}^{k} + \tau _{{{\rm{xz}}}}^{k} {\rm{\delta \gamma }}_{{{\rm{xz}}}}^{k} + \tau _{{{\rm{yz}}}}^{k} {\rm{\delta \gamma }}_{{{\rm{yz}}}}^{k} } \right)dzdxdy;$$

By assuming uniform applied load (*p*) in the z-direction, the work of an external force can be obtained using:

10 $${\delta W} = \mathop \smallint \limits_{A} \left( {p{\updelta }w} \right)dxdy$$

By replacing Eqs. (4) and (5) in Eq. (9) and also replacing Eqs. (9) and (10) in Eq. (8) and integrating through the thickness of the plate, Eq. (8) can be rewritten as:

11 $$\begin{gathered} \int\limits_{A} {\left( {\left( {N_{xx} \left( {\frac{\partial \delta u}{{\partial x}}} \right) + M_{xx} \left( {\frac{{\partial \delta \theta_{x} }}{\partial x}} \right) + M_{xx}^{\phi } \frac{{\partial \delta \psi_{x} }}{\partial x} + N_{yy} \left( {\frac{\partial \delta v}{{\partial y}}} \right) + M_{yy} \left( {\frac{{\partial \delta \theta_{y} }}{\partial y}} \right)} \right.} \right.} \hfill \\ + M_{yy}^{\phi } \left( {\frac{{\partial \delta \psi_{y} }}{\partial y}} \right) + M_{yy}^{\phi } \frac{{\partial \psi_{y} }}{\partial y} + N_{xy} \left( {\frac{\partial \delta u}{{\partial y}} + \frac{\partial \delta v}{{\partial x}}} \right) \hfill \\ + M_{xy} \left( {\frac{{\partial \delta \theta_{x} }}{\partial y} + \frac{{\partial \delta \theta_{y} }}{\partial x}} \right) + M_{xy}^{\phi } \frac{{\partial \delta \psi_{x} }}{\partial y} + M_{yx}^{\phi } \frac{{\partial \delta \psi_{y} }}{\partial x} \hfill \\ \left. {\left( { + Q_{xz} \left( {\frac{\partial \delta w}{{\partial x}} + \delta \theta_{x} } \right) + Q_{xz}^{\phi } \delta \psi_{x} + Q_{yy} \left( {\frac{\partial \delta w}{{\partial y}} + \delta \theta_{y} } \right) + Q_{yz}^{\phi } \delta \psi_{y} } \right)} \right) \hfill \\ \left. { - \left( {p\delta w} \right)} \right)dA = 0 \hfill \\ \end{gathered}$$

12 $$\begin{gathered} \left( {N_{{{\text{xx}}}} ,N_{{{\text{yy}}}} ,N_{{{\text{xy}}}} } \right) = \mathop \smallint \limits_{ - h}^{h} \left( {\sigma_{{{\text{xx}}}}^{k} , \sigma_{{{\text{yy}}}}^{k} ,\sigma_{{{\text{xy}}}}^{k} } \right){\text{d}}z \hfill \\ \left( {M_{{{\text{xx}}}} ,M_{{{\text{yy}}}} ,M_{{{\text{xy}}}} } \right) = \mathop \smallint \limits_{ - h}^{h} \left( {\sigma_{{{\text{xx}}}}^{k} , \sigma_{{{\text{yy}}}}^{k} , \sigma_{{{\text{xy}}}}^{k} } \right)z{\text{d}}z \hfill \\ \left( {Q_{{{\text{xz}}}} ,Q_{{{\text{yz}}}} } \right) = \mathop \smallint \limits_{ - h}^{h} \left( {\sigma_{{{\text{xz}}}}^{k} , \sigma_{{{\text{yz}}}}^{k} } \right){\text{d}}z \hfill \\ \left( {M_{xx}^{\phi } ,M_{yy}^{\phi } ,M_{xy}^{\phi } ,M_{yx}^{\phi } } \right) = \mathop \smallint \limits_{ - h}^{h} \left( {\phi_{x}^{k} \sigma_{{{\text{xx}}}}^{k} , \phi_{y}^{k} \sigma_{{{\text{yy}}}}^{k} , \phi_{x}^{k} \sigma_{{{\text{xy}}}}^{k} , \phi_{y}^{k} \sigma_{{{\text{xy}}}}^{k} } \right){\text{d}}z \hfill \\ \left( {Q_{xz}^{\phi } ,Q_{yz}^{\phi } } \right) = \mathop \smallint \limits_{ - h}^{h} \left( {\frac{{\partial \phi_{x}^{k} }}{\partial z}\sigma_{{{\text{xz}}}}^{k} , \frac{{\partial \phi_{y}^{k} }}{\partial z}\sigma_{{{\text{yz}}}}^{k} } \right){\text{d}}z \hfill \\ \end{gathered}$$

The $\phi$ indices denote to the zig-zag functions, ${N}_{ij}$ and ${Q}_{iz}$ are in-plane and out-of-plane forces, and ${M}_{ij}$ represent bending moments which can be calculated as follow:

By applying integration by parts to Eq. (12) and calculating variation coefficients, the governing equations of the sandwich panel can be obtained in the form of Eq. (13).

13 $$\begin{gathered} {\rm{\delta U}}:~N_{{xx,x}} + N_{{xy,y}} = 0 \hfill \\ {\rm{\delta V}}:~N_{{{\rm{yy}},y}} + N_{{{\rm{xy}},x}} = 0 \hfill \\ {\rm{\delta W}}:~Q_{{{\rm{xz}},x}} + Q_{{{\rm{yz}},y}} - w = 0 \hfill \\ {\updelta \uptheta}_{x} :{\rm{~M}}_{{xx,x}} + M_{{xy,y}} - Q_{{{\rm{xz}}}} = 0 \hfill \\ {\updelta \uptheta}_{y} :{\rm{~M}}_{{{\rm{yy}},y}} + M_{{{\rm{xy}},x}} - Q_{{{\rm{yz}}}} = 0 \hfill \\ {\updelta \uppsi}_{x} :~M_{{xx,x}}^{\phi } + M_{{xy,y}}^{\phi } - Q_{{xz}}^{\phi } = 0 \hfill \\ {\updelta \uppsi}_{y} :M_{{yy,y}}^{\phi } + M_{{yx,x}}^{\phi } - Q_{{yz}}^{\phi } = 0 \hfill \\ \end{gathered}$$

We used Galerkin method to solve the differential governing equation system for a simply supported sandwich plate. Assuming a quasi-static condition, the unknown functions are considered as Eq. (14).

14 $$\begin{gathered} {\rm{u}}\left( {{\rm{x,y}}} \right) = \mathop \sum \limits_{{{\rm{n}} = 1}}^{{\rm{N}}} \mathop \sum \limits_{{{\rm{m}} = 1}}^{{\rm{M}}} {\rm{u}}_{{{\rm{m,n}}}} \overline{\overline{{\rm{u}}}} \left( {{\rm{x,y}}} \right) \hfill \\ {\rm{v}}\left( {{\rm{x,y}}} \right) = \mathop \sum \limits_{{{\rm{n}} = 1}}^{{\rm{N}}} \mathop \sum \limits_{{{\rm{m}} = 1}}^{{\rm{M}}} {\rm{v}}_{{{\rm{m,n}}}} \overline{\overline{{\rm{v}}}} \left( {{\rm{x,y}}} \right) \hfill \\ {\rm{w}}\left( {{\rm{x,y}}} \right) = \mathop \sum \limits_{{{\rm{n}} = 1}}^{{\rm{N}}} \mathop \sum \limits_{{{\rm{m}} = 1}}^{{\rm{M}}} {\rm{w}}_{{{\rm{m,n}}}} \overline{\overline{{\rm{w}}}} \left( {{\rm{x,y}}} \right) \hfill \\ {\uptheta}_{{\rm{x}}} \left( {{\rm{x,y}}} \right) = \mathop \sum \limits_{{{\rm{n}} = 1}}^{{\rm{N}}} \mathop \sum \limits_{{{\rm{m}} = 1}}^{{\rm{M}}} {\uptheta}_{{{\rm{x}}_{{{\rm{m,n}}}} }} \overline{\overline{{{\uptheta}_{{\rm{x}}} }}} \left( {{\rm{x,y}}} \right) \hfill \\ {\uptheta}_{{\rm{y}}} \left( {{\rm{x,y}}} \right) = \mathop \sum \limits_{{{\rm{n}} = 1}}^{{\rm{N}}} \mathop \sum \limits_{{{\rm{m}} = 1}}^{{\rm{M}}} {\uptheta}_{{{\rm{y}}_{{{\rm{m,n}}}} }} \overline{\overline{{{\uptheta}_{{\rm{y}}} }}} \left( {{\rm{x,y}}} \right) \hfill \\ {\uppsi}_{{\rm{x}}} \left( {{\rm{x,y}}} \right) = \mathop \sum \limits_{{{\rm{n}} = 1}}^{{\rm{N}}} \mathop \sum \limits_{{{\rm{m}} = 1}}^{{\rm{M}}} {\uppsi}_{{{\rm{x}}_{{{\rm{m,n}}}} }} \overline{\overline{{{\uppsi}_{{\rm{x}}} }}} \left( {{\rm{x,y}}} \right) \hfill \\ {\uppsi}_{{\rm{y}}} \left( {{\rm{x,y}}} \right) = \mathop \sum \limits_{{{\rm{n}} = 1}}^{{\rm{N}}} \mathop \sum \limits_{{{\rm{m}} = 1}}^{{\rm{M}}} {\uppsi}_{{{\rm{y}}_{{{\rm{m,n}}}} }} \overline{\overline{{{\uppsi}_{{\rm{y}}} }}} \left( {{\rm{x,y}}} \right) \hfill \\ \end{gathered}$$

${u}_{m,n}$, ${v}_{m,n}$, ${w}_{m,n}$,${{\uptheta }_{\mathrm{x}}}_{\mathrm{m}\text{,n}}$, ${{\uptheta }_{\mathrm{y}}}_{\mathrm{m}\text{,n}}$, ${{\uppsi }_{\mathrm{x}}}_{\mathrm{m}\text{,n}}$ and ${{\uppsi }_{\mathrm{y}}}_{\mathrm{m}\text{,n}}$ are unknown constants that can be obtained by minimizing the error. $\overline{\overline{u}} \left( {x{\text{,y}}} \right)$, $\overline{\overline{v}} \left( {x{\text{,y}}} \right)$, $\overline{\overline{w}} \left( {x{\text{,y}}} \right)$, $\overline{\overline{{{\uptheta }_{x} }}} \left( {x{\text{,y}}} \right)$, $\overline{\overline{{{\uptheta }_{y} }}} \left( {x{\text{,y}}} \right)$, $\overline{\overline{{\psi_{x} }}} \left( {x{\text{,y}}} \right)$ and $\overline{\overline{{ \psi_{y} }}} \left( {x{\text{,y}}} \right)$ are test functions that should satisfy the minimum necessary boundary conditions. For the simply supported boundary condition, the test functions can be recalculated in the form of:

15 $$\left\{ \begin{gathered} \overline{\overline{u}} \left( {x{\text{,y}}} \right) = \cos \left( {\frac{m\pi x}{a}} \right)\sin \left( {\frac{n\pi y}{b}} \right) \hfill \\ \overline{\overline{v}} \left( {x{\text{,y}}} \right) = \sin \left( {\frac{m\pi x}{a}} \right)\cos \left( {\frac{n\pi y}{b}} \right) \hfill \\ \overline{\overline{w}} \left( {x{\text{,y}}} \right) = \sin \left( {\frac{m\pi x}{a}} \right)\sin \left( {\frac{n\pi y}{b}} \right) \hfill \\ \overline{\overline{{{\uptheta }_{x} }}} \left( {x{\text{,y}}} \right) = \cos \left( {\frac{m\pi x}{a}} \right)\sin \left( {\frac{n\pi y}{b}} \right) \hfill \\ \overline{\overline{{{\uptheta }_{y} }}} \left( {x{\text{,y}}} \right) = \sin \left( {\frac{m\pi x}{a}} \right)\cos \left( {\frac{n\pi y}{b}} \right) \hfill \\ \overline{\overline{{\psi_{x} }}} \left( {x{\text{,y}}} \right) = \cos \left( {\frac{m\pi x}{a}} \right)\sin \left( {\frac{n\pi y}{b}} \right) \hfill \\ \overline{\overline{{\psi_{y} }}} \left( {x{\text{,y}}} \right) = \sin \left( {\frac{m\pi x}{a}} \right)\cos \left( {\frac{n\pi y}{b}} \right) \hfill \\ \end{gathered} \right.$$

A system of algebraic equations is obtained by substituting Eq. (14) to governing equations, which can lead to achieving the unknown coefficients in Eq. (14).

We used finite element modelling (FEM) approaches to computationally simulate the flexural bending of simply supported sandwich panels with a re-entrant lattice structure as a core. The analyses were performed in a commercial finite element code (*i.e.*, Abaqus ver 6.12.1). A three-dimensional hexahedral solid element with a reduced integration (C3D8R) was used for modelling the top and bottom layers and a linear tetrahedron element (C3D4) was used to model the middle (re-entrant) lattice structure. We performed a mesh sensitivity analysis to check the mesh convergency and concluded that the displacement results converged by an element size equal to the minimum thickness among three layers. The sandwich plate was loaded using a sinusoidal loading function while a simply supported boundary condition was considered on four edges. A linear elastic mechanical behavior was considered as the material model which was assigned to all layers. No contact was defined between layers and they were tied together.

## Experimental tests

We used 3D printing techniques to create our prototypes (*i.e.*, sandwich plate with auxetic core printed three times) and the corresponding custom-made experimental setup to apply similar flexural bending (uniform load *p* along the z-direction) and boundary conditions (*i.e.*, simply support) assumed in our analytical approach (Fig. 1).

The 3D printed sandwich panel, here, was composed of two skins (top and bottom) and a re-entrant lattice core whose dimensions are listed in Table 1 and were fabricated using a Ultimaker 3 (Italy) 3D printing machine that uses Fused Deposition Modeling (FDM) technique for its processes. We 3D printed the bottom plate and the core auxetic lattice structures together while we 3D printed the top layer separately. This helped to avoid any complexity for the support removal processes in case the entire structure was supposed to be printed at once. Once two separate parts were 3D printed, they were glued together using a super glue. We used polylactic acid (PLA) for printing these components and set the infill density to the maximum (*i.e.*, 100%) to prevent any local defects while printing.

**Table 1:** The dimensions of the 3D printed sandwich panel prototypew with an auxetic core.

*a*[*mm*]	*b*[*mm*]	*h*_*t*_[*mm*]	*h*_*c*_[*mm*]	*h*_*b*_[*mm*]	*L*[*mm*]	*θ*[*deg*]	*t*[*mm*]	*h*[*mm*]
70	70	0*.*5	3	0*.*5	5*.*7	70	1*.*3	10

**Figure 2:**
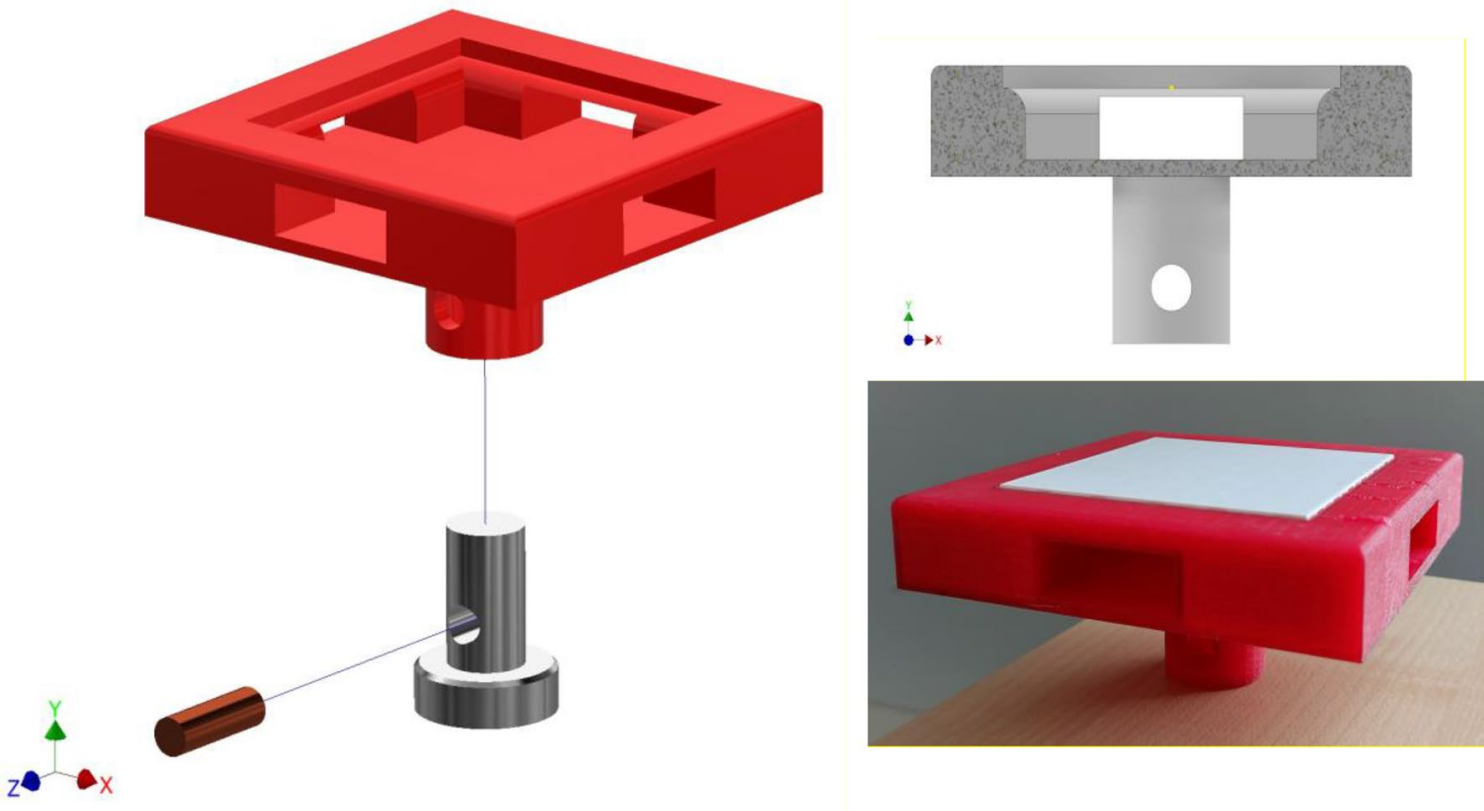
The schematic of the 3D printed custom-made gripping system and their assemblage with the 3D printed sandwich panel with an auxetic core.

The custom-made gripping system mimicked the similar simply support boundary conditions assumed in our analytical models. This means that the gripping system disabled the displacement of the plate in the x- and y- directions along its edges while allowing the free-rotation of those edges about the x- and y- axes. This has been performed by considering a fillet with a radius of *r* = *h/*2 at the four edges of the gripping system (Fig. 2). This gripping system also ensured that the applied load was fully transferred from the testing machine to the plate, and it was in line with the center line of the plate (Fig. 2). We used polyjet 3D printing technique (ObjetJ735 Connex3, Stratasys® Ltd., USA) with a hard commercial polymer (*i.e.,* Vero family) for printing the gripping system.

We performed displacement-control quasi-static compression tests using a mechanical test benchmark (Lloyd LR, load cell = 100 N), and collected the force and displacement from the machine at the sampling rate of 20 Hz.

## Results and discussion

The numerical study of the proposed sandwich structure is presented in this section. We assumed that the top and bottom layers were made of carbon epoxy, and the re-entrant core lattice structure was made of polymer. The mechanical properties of the materials used in this study are shown in Table 2. Also, the dimensionless relations of displacement and stress field results are given in Table 3.

**Table 2:** The mechanical properties of layers (GPa).

Properties	GFRP	Polymer
${E}_{11}^{k}$	62	0.104
${{E}_{22}^{k}=E}_{33}^{k}$	4.8	
${v}_{12}^{k}={v}_{13}^{k}$	0.22	0.3
${v}_{23}^{k}$	0.3	
${G}_{12}^{k}={G}_{13}^{k}$	3.27	
${G}_{23}^{k}$	1.8

**Table 3:** Dimensionless relations of mechanical parameters.

Parameter	Dimensionless relation
$\overline{{\varvec{W}} }$	${{\varvec{W}}\times 10}^{3}\mathbf{D}11/\left(\mathbf{p}{{\varvec{a}}}^{4}\right)({\varvec{a}}/2, {\varvec{b}}/2)$
${\overline{\upsigma } }_{xx}$	${{\varvec{\upsigma}}}_{{\varvec{x}}{\varvec{x}}}\times (4{{\varvec{h}}}^{2})/(\mathbf{p}{{\varvec{a}}}^{2})({\varvec{a}}/2, {\varvec{b}}/2)$
${\overline{{\varvec{\uptau}}} }_{xy}$	${{\varvec{\uptau}}}_{{\varvec{x}}{\varvec{y}}}\times ({{\varvec{h}}}^{2})/(\mathbf{p}{{\varvec{a}}}^{2})(0, 0)$
${\overline{{\varvec{\uptau}}} }_{xz}$	${{\varvec{\uptau}}}_{{\varvec{x}}{\varvec{z}}}\times (2{\varvec{h}})/(\mathbf{p}{\varvec{a}})(0, {\varvec{b}}/2)$
${\overline{{\varvec{\uptau}}} }_{yz}$	${{\varvec{\uptau}}}_{{\varvec{y}}{\varvec{z}}}\times (2{\varvec{h}})/(\mathbf{p}{\varvec{a}})({\varvec{a}}/2,0)$
$\overline{\mathcal{Z} }$	z/h

The maximum vertical dimensionless displacement for the simply supported plate with a uniform loading is compared with those obtained from different methods (Table 4). There is a good agreement between the presented theory, FEM, and experimental test.

**Table 4:** The maximum vertical dimensionless displacement, $\overline{W }$, for a simply supported sandwich plate with an auxetic core under a uniform compressive loading.

Method	Max. Disp.
Experiment	2.6760
FEM	2.3854
Presented theory	2.2692

**Figure 3:**
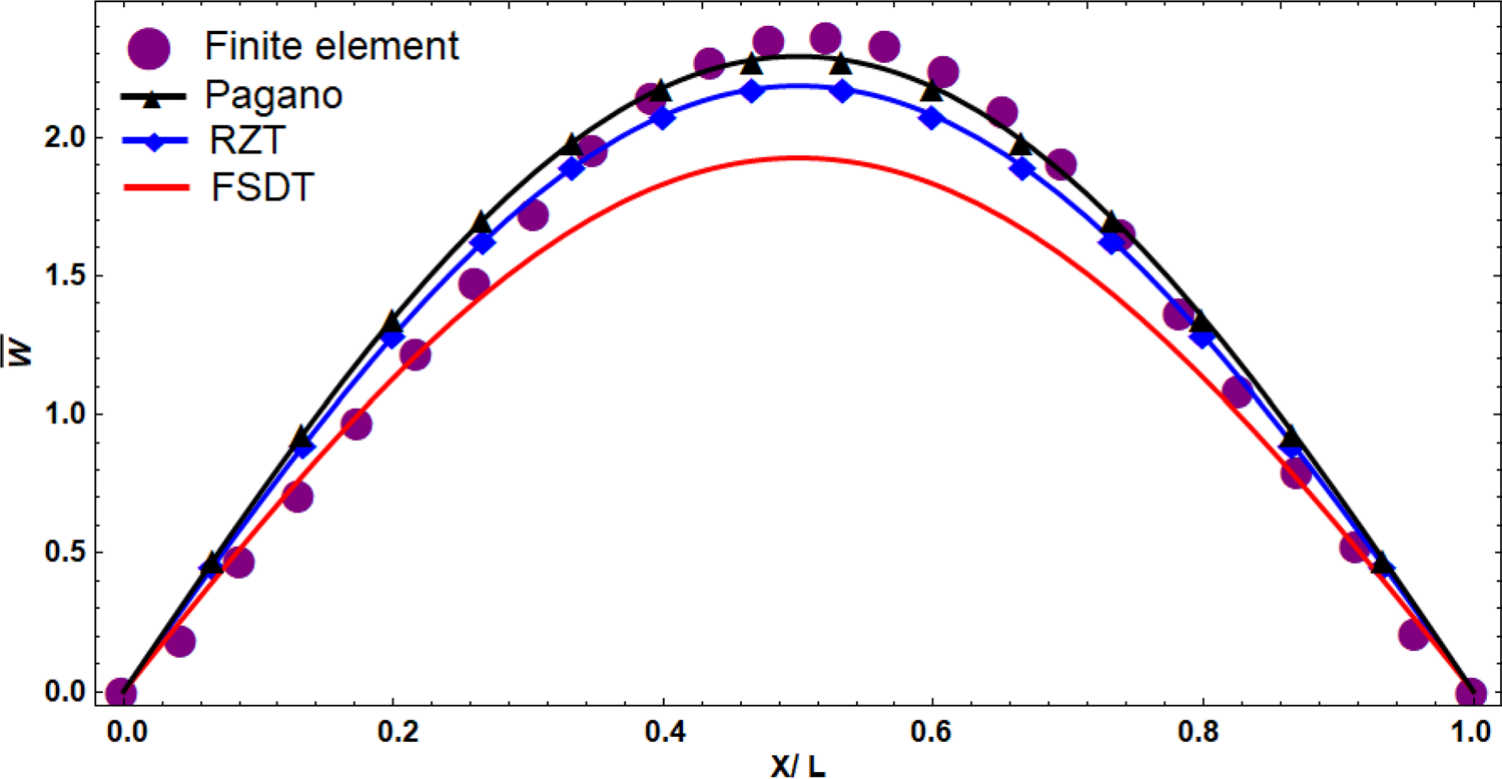
The comparison of the normalized vertical deformation calculated using different theories at y = b/2.

**Figure 4:**
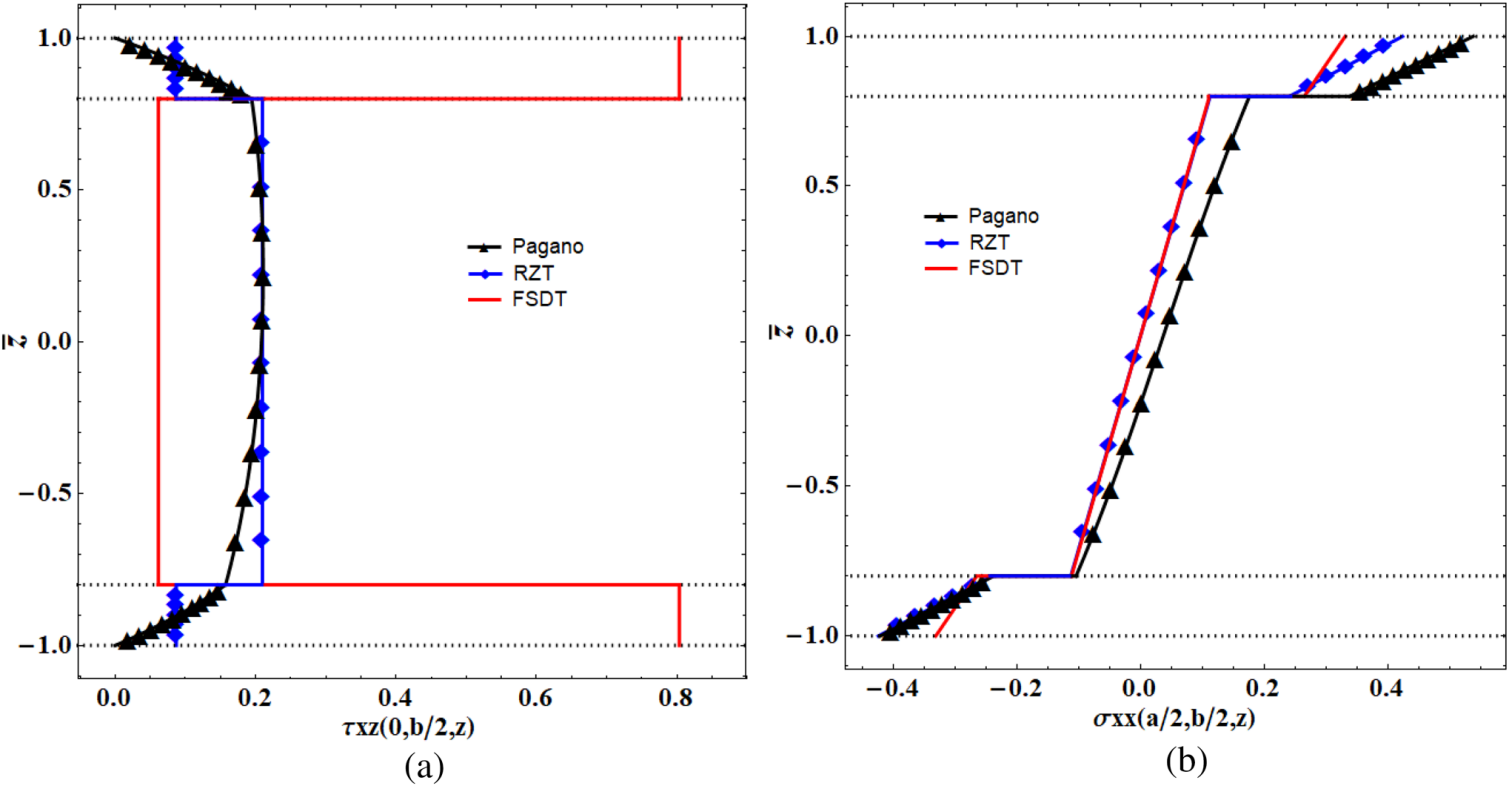
The variation of (**a**) shear stress, and (**b**) normal stress, along the thickness of the sandwich panel calculated using different theories.

We compared the vertical displacement of the refined zig-zag theory (RZT) with the 3D elasticity theory (Pagano), the first-order shear deformation theory (FSDT), and FEM results (see Fig. 3). According to the displacement plots of a thick sandwich plate, the first-order shear deformation theory had the maximum difference with the elasticity solution. The refined zig-zag theory, however, predicted the highly accurate result. Furthermore, we compared the out-of-plane shear stress and the in-plane normal stress of different theories in which the zig-zag theory reached more accurate results than FSDT (Fig. 4).

**Figure 5:**
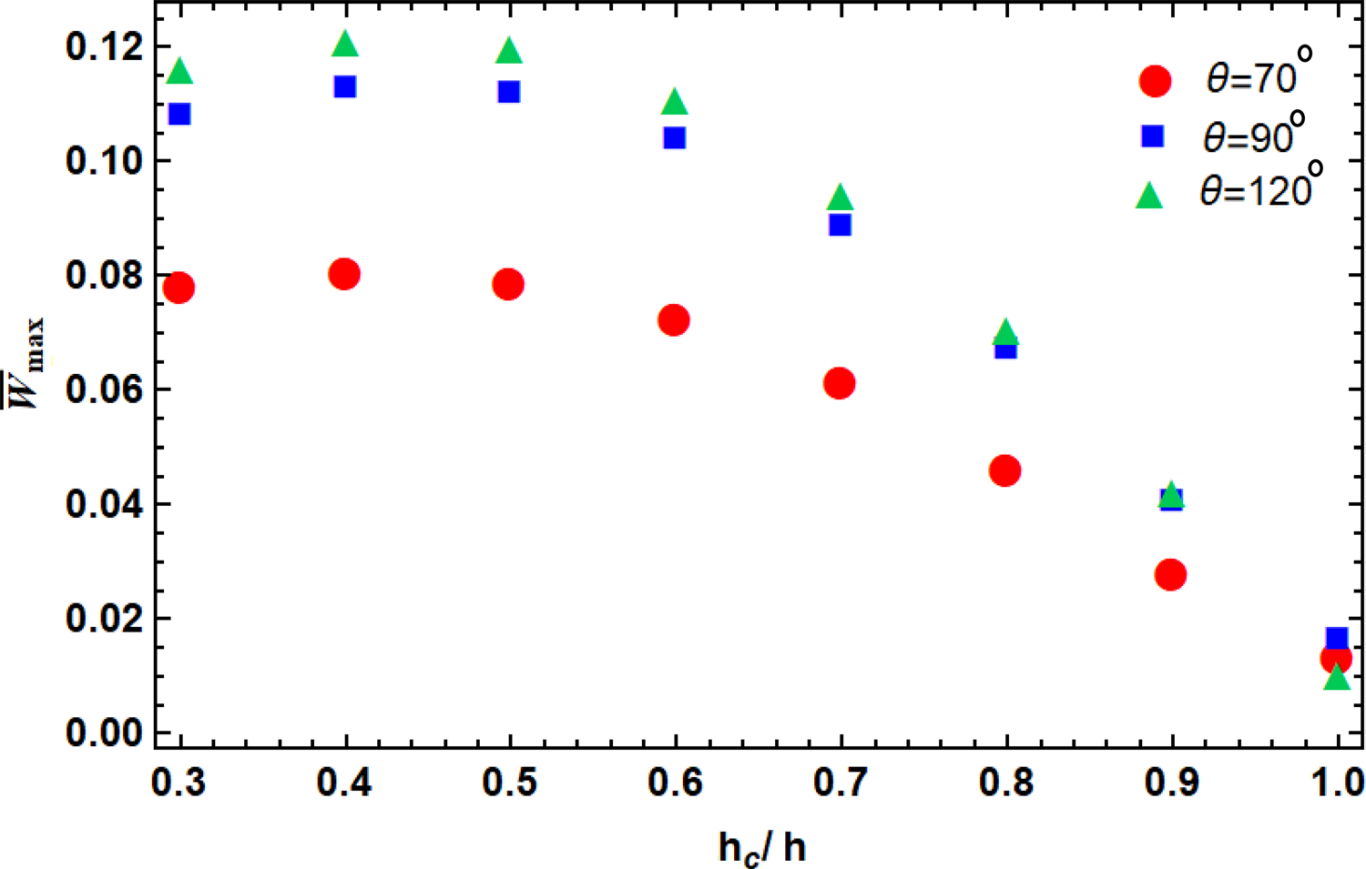
The normalized maximum deflection of re-entrant lattice core with different unit cell angles and out-of-plane thicknesses.

We further analyzed the effects of the geometrical parameters of the unit cells of the re-entrant core on the overall mechanical properties of the sandwich plate. The angle of the unit cells is the most important geometrical parameter in the design of re-entrant lattice structures^34–36^. We, therefore, calculated the effects of the angle of unit cells as well as the out-of-plane thickness of the core layer on the overall deflection of the plate (Fig. 5). The maximum dimensionless deflection decreased by increasing the thickness of the intermediate layer. The relative flexural strength increased for a thicker core layer and when $\frac{{h}_{c}}{h}=1$ (i.e., when there is a single re-entrant layer). The sandwich panel with an auxetic unit cell (*i.e.*, $\theta =70^\circ$), had the lowest displacement (Fig. 5). This indicates that the flexural strength of the auxetic core is more than the conventional one, is less effective and there in positive Poisson ratios.

**Figure 6:**
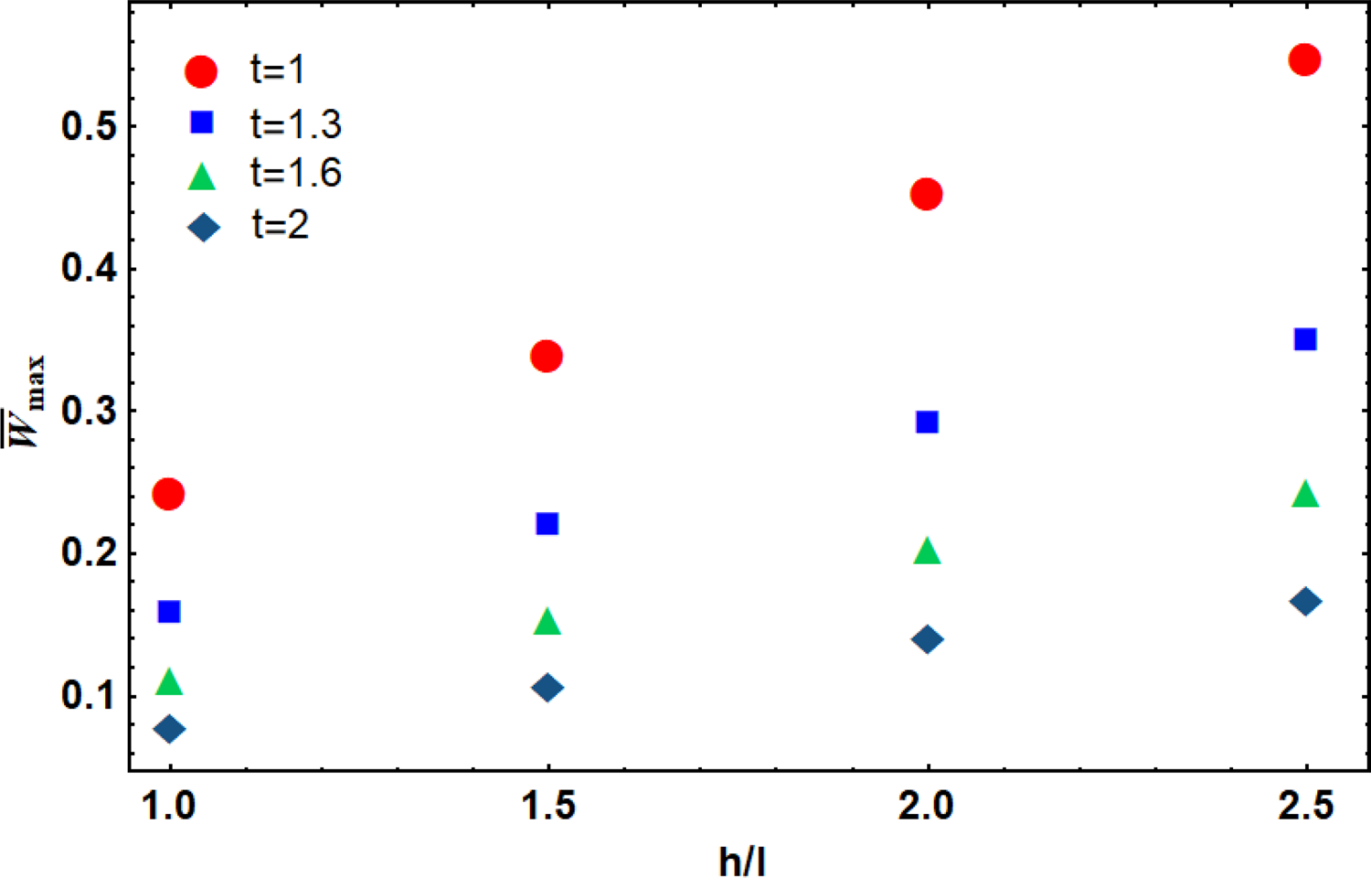
The maximum deflection of the sandwich plate by different thicknesses and lengths of an auxetic core lattice structure.

The thickness and length-to-height ratio of an auxetic lattice core (*i.e.*, $\theta =70^\circ$) influenced the maximum displacement of the sandwich plate (Fig. 6). As it can be observed, the maximum deflection of the plate increased by increasing *h/l*. Besides, increasing the thickness of the auxetic core reduced the porosity of the re-entrant structure, which increased the bending flexural strength of the structure.

**Figure 7:**
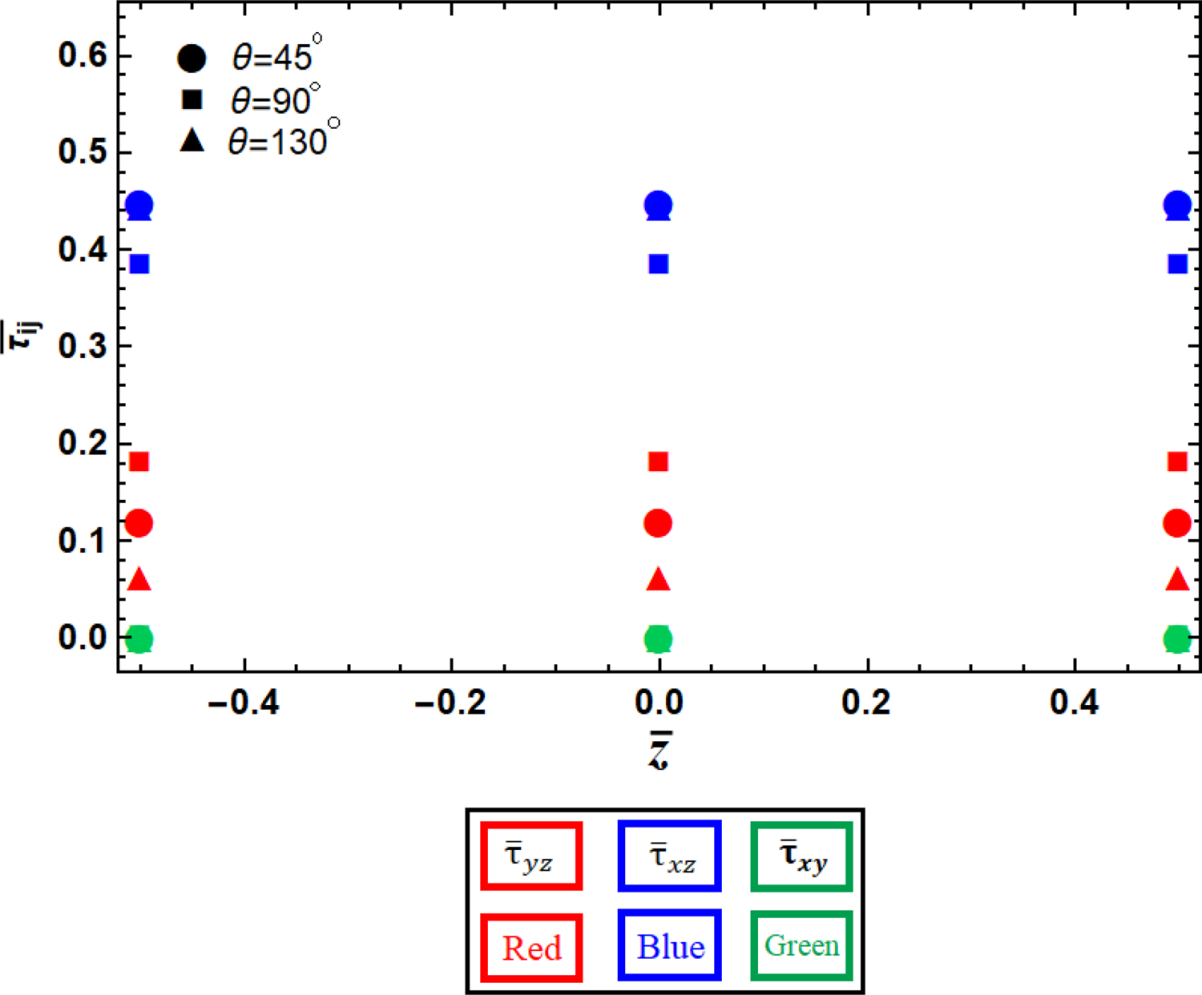
The variation of the components of the shear stresses at different layers of sandwich panels with various re-entrant lattice cores.

The study of the stress field is an interesting area that can be explored by changing the geometrical parameters of the unit cells, thereby, investigating the failure modes (*e.g.*, delamination) of the sandwich structures. The values of Poisson’s ratio have a larger effect on the out-of-plane shear stress field than normal stresses (see Fig. 7). Furthermore, this effect is not consistent in different directions since those lattices have orthotropic material properties. The other geometrical parameters such as thickness, height, and length of the re-entrant structure had a negligible effect on the stress field and, therefore, they have not been analyzed in this study.

## Conclusion

Here, the bending flexural strength of a simply supported sandwich plate with a re-entrant lattice core was investigated by zig-zag theory. The presented formulation was compared with other classical theories including the 3D elasticity, first-order shear deformation theory, and FEM. We also verified our approach by comparing our results with experimental results of the 3D printed sandwich structures. Our results showed that the zig-zag theory was capable of predicting the deformation of moderately thick sandwich structures under the flexural bending loading. Moreover, the effects of the geometrical parameters of the re-entrant lattice structures on the bending behavior of the sandwich plates were analyzed. The results showed that by increasing the level of auxeticity (*i. e.*, θ < 90), the flexural bending strength increased. Also, increasing the length-to-height ratio and decreasing the thickness of the core lattice reduced the bending flexural strength of the sandwich plate. In the end, the effect of the Poisson’s ratio on the out-of-plane shear stresses was studied which confirmed that it had the greatest effect on the shear stresses created along with the thickness of those sandwich plates. The presented formulation and conclusion can pave ways in designing and optimizing sandwich structures with a re-entrant lattice core under more complex loading conditions necessary for in the design of load-bearing structures in aerospace and biomedical engineering.

## Data Availability

The datasets used and/or analysed during the current study available from the corresponding author on reasonable request.
